# Evolutionary insights into heart regeneration

**DOI:** 10.1186/s13619-020-00069-x

**Published:** 2020-12-01

**Authors:** Jing-Wei Xiong

**Affiliations:** Beijing Key Laboratory of Cardiometabolic Molecular Medicine, Institute of Molecular Medicine, and State Key Laboratory of Natural and Biomimetic Drugs, Peking University, Beijing, 100871 China

## Abstract

Some lower vertebrates such as zebrafish and axolotl have incredible cardiac regenerative potential while mammals have very limited ones. Comparative studies among species have revealed that cardiomyocyte polyploidy, endothermy, and injury-induced activation of certain transcriptional factors including AP1 complexes are critical for cardiomyocyte proliferation and heart regeneration during animal evolution. Gaining insights into these evolutionarily conserved mechanisms will likely lead to achieving heart regeneration in non-regenerative mammals including humans.

Regenerative potential in the animal kingdom is a fundamental topic of regenerative biology and medicine. With the development of genome science and genetics technology, many investigators started to address how regenerative potential is lost or gained during the evolutionary courses of particular animal species. It is now documented that either whole-animal or organ regeneration is achieved by activating adult stem cells (such as hydra and planarians; blood, skins, skeletal muscles, and livers in vertebrates), by inducing Muller glia reprogramming into neurons in adult zebrafish retinas, or by promoting cardiomyocyte (CM) proliferation in adult zebrafish hearts, and neonatal mouse, rat, and pig hearts (Beisaw et al. 2020; Duncan and Sanchez Alvarado 2019; Goldman and Poss 2020; Hoang et al. 2020; Tzahor and Poss 2017; Wang et al. 2020). In this short opinion article, we review several recent studies on evolutionary insights into heart regeneration that may be exploited for promoting non-regenerative heart regeneration.

Most mammalian cardiomyocytes (CMs) are mononuclear diploid CMs during development, but they become mostly polyploid CMs at late gestation or early postnatal stages. The appearance of CM polyploidy conincides with the loss of cardiac regenerative potential in mice, rats, and pigs (Gan et al. 2020). However, it remains largely unknown whether CM polyploidy is a causative factor for the loss of cardiac regenerative potential. A recent work has reported that the frequency of adult mononuclear diploid cardiomyocytes (MDCMs) is highly correlated to the cardiac regenerative potential in a large collection of 120 inbred mouse strains (Patterson et al. 2017). The authors meticulously assessed the percentage of MDCMs in 120 mouse strains, ranging from 2% to 18% MDCMs, and they then found that the more MDCMs, the better their hearts regenerate, with increased CM proliferation after myocardial infarction (MI). Genome-wide association analysis has revealed that *Tnni3k* is associated with this phenotype, which is further supported by assessing the percentage of MDCMs, CM proliferation, and the distribution of diploid and polyploid CMs in cardiac-specific *Tnni3k* knockout mice after MI. Nevertheless, they did not find improvement in cardiac function and fibrosis in these mutant mice compared with control siblings post MI. On the other hand, they found that CMs are primarily diploids in zebrafish and cardiac-specific overexpression of zebrafish *tnni3k* leads to more CM polyploidy and loss of their regenerative capacity after ventricular resection. Similarly, transient inhibition of cytokinesis by cardiac-specific overexpression of dominant-negative Ect2 results in more CM polyploidy and compromised cardiac regeneration in zebrafish (Gonzalez-Rosa et al. 2018). Together, these works present new mechanistic insights into the causative effect of CM polyploidy on heart regeneration.

Another elegant work has reported that the loss of cardiac regenerative potential is highly related with CM polyploidization via phylogenetic analysis of CM nucleation and polyploidy in a large collection of non-vertebrates and vertebrates, and the percentage of MDCMs inversely correlates with metabolic rate, body temperature, and serum total thyroid hormone T4 levels (Hirose et al. 2019). They have further demonstrated that cardiac-specific inhibition of thyroid hormone receptor a (*Thra*) increases CM proliferation and the percentage of MNCMs in neonatal and adult mice, and results in an evident improvement in cardiac function and fibrosis after myocardial ischemic reperfusion in adult mice. Consistently, exogenous application of thyroid hormone T3 inhibits heart regeneration with evident CM polyploidization and decreased CM proliferation in zebrafish. Thus, thyroid hormone signaling promotes CM polyploidy and decreases cardiac regenerative potential during animal evolution.

As illustrated above, cardiac regenerative potential inversely correlates with CM polyploidization and postnatal endothermy/body temperature during animal evolution. While having learned a great deal of signaling pathways in regulating CM proliferation and heart regeneration, we still have very limited knowledge on the roles of non-coding DNA elements in heart regeneration, which are quite diverse during evolutionary species. Comparing with previous studies on searching for regenerative enhancers from different organ regeneration processes in zebrafish (Goldman et al. 2017; Kang et al. 2016; Thompson et al. 2020), Sánchez Alvarado and colleagues have recently reported the isolation of 49 conserved regenerative enhancers in the hearts and fins between killifish and zebrafish, which a good fraction of these 49 enhancers are also conserved in regenerative mouse strain *acomys cahirinus* but not non-regenerative mouse strain *mus musculus* (Wang et al. 2020). They have reported that the enhancer inhba is highly conserved between regenerative zebrafish (Z-IEN) and killifish (K-IEN) but not in non-regenerative humans (H-IEN), and this enhancer is activated in the progenitors of blastema during fin regeneration. Genetic depletion of this enhancer in killifish (K-IEN) suggests its essential roles in both heart and fin regeneration. Further bioinformatics analysis identified that AP-1 binding sites are enriched in these 49 regenerative enhancers and are also essential for regeneration. Together with other works (Beisaw et al. 2020; Gehrke et al. 2019), the AP-1 complexes appear to be evolutionarily conserved and injury-induced transcription factors for heart regeneration.

In brief, these recent studies have utilized comparative studies on heart regeneration among evolutionary species, leading to identifying critical factors such as thyroid hormones and cytokinesis factor (Tnni3k) for regulating CM nucleation and polyploidization, as well as non-coding DNA elements (regenerative enhancers) and binding factors (AP-1 complexes) for regulating CM proliferation. These and future studies will likely reveal a network of transcription factors and enhancers for coordinating CM proliferation and heart regeneration. In addition to these critical factors and regenerative enhancers, the field will take advantage of genome editing and chemical biology approaches to identify factors and small molecules that are sufficient for promoting non-regenerative heart regeneration in the coming years.
